# High prevalence of asymptomatic malaria infections in adults, Ashanti Region, Ghana, 2018

**DOI:** 10.1186/s12936-020-03441-z

**Published:** 2020-10-12

**Authors:** Melina Heinemann, Richard O. Phillips, Christof D. Vinnemeier, Christina C. Rolling, Egbert Tannich, Thierry Rolling

**Affiliations:** 1 Division of Infectious Diseases, I. Department of Medicine, University Medical Centre Hamburg-Eppendorf, Hamburg, Germany; 2 Department of Tropical Medicine, Bernhard Nocht Institute for Tropical Medicine, Hamburg, Germany; 3 Kumasi Center for Collaborative Research in Tropical Medicine, Kumasi, Ghana; 4 Department of Medicine, University Medical Centre Hamburg-Eppendorf, Hamburg, Germany; 5 National Reference Centre for Tropical Pathogens, Bernhard Nocht Institute for Tropical Medicine, Hamburg, Germany; 6 German Centre for Infection Research (DZIF), Partner Site Hamburg-Lübeck-Borstel-Riems, Hamburg, Germany; 7 Department of Clinical Immunology of Infectious Diseases, Bernhard Nocht Institute for Tropical Medicine, Hamburg, Germany; 8 Present Address: Weizmann Institute of Science, Rehovot, Israel; 9 Present Address: NYU Langone Medical Center, New York, NY USA; 10 Present Address: Infectious Disease Service, Department of Medicine, Memorial Sloan Kettering Cancer Center, New York, NY USA

**Keywords:** Asymptomatic malaria, *Plasmodium falciparum*, *Plasmodium malariae*, *Plasmodium ovale curtisi*, *Plasmodium ovale wallikeri*, Ghana, Sub-Saharan Africa, Rapid diagnostic test, Polymerase chain reaction, Molecular prevalence

## Abstract

**Background:**

Ghana is among the high-burden countries for malaria infections and recently reported a notable increase in malaria cases. While asymptomatic parasitaemia is increasingly recognized as a hurdle for malaria elimination, studies on asymptomatic malaria are scarce, and usually focus on children and on non-falciparum species. The present study aims to assess the prevalence of asymptomatic *Plasmodium falciparum* and non-falciparum infections in Ghanaian adults in the Ashanti region during the high transmission season.

**Methods:**

Asymptomatic adult residents from five villages in the Ashanti Region, Ghana, were screened for *Plasmodium* species by rapid diagnostic test (RDT) and polymerase chain reaction (PCR) during the rainy season. Samples tested positive were subtyped using species-specific real-time PCR. For all *Plasmodium ovale* infections additional sub-species identification was performed.

**Results:**

Molecular prevalence of asymptomatic *Plasmodium* infection was 284/391 (73%); only 126 (32%) infections were detected by RDT. While 266 (68%) participants were infected with *Plasmodium falciparum,* 33 (8%) were infected with *Plasmodium malariae* and 34 (9%) with *P. ovale.* The sub-species *P. ovale curtisi* and *P. ovale wallikeri* were identified to similar proportions. Non-falciparum infections usually presented as mixed infections with *P. falciparum.*

**Conclusions:**

Most adult residents in the Ghanaian forest zone are asymptomatic *Plasmodium* carriers. The high *Plasmodium* prevalence not detected by RDT in adults highlights that malaria eradication efforts must target all members of the population. Beneath *Plasmodium falciparum,* screening and treatment must also include infections with *P. malariae*, *P. o. curtisi* and *P. o. wallikeri*.

## Background

The World Health Organization (WHO) aims to achieve a global reduction in malaria case incidence and mortality rates by 90% compared to 2015, elimination in at least 35 countries, and prevention of reintroduction in all malaria-free countries by the year 2030. Although the worldwide burden of malaria substantially decreased since 2010, some high-burden countries in Africa report an increase in malaria cases. According to the WHO, the global targets for 2030 will not be achieved unless there is accelerated change. Ghana belongs to the eleven high-burden countries accounting for > 70% of the global malaria cases and deaths. With an increase of malaria cases by 8%, Ghana is among the two highest burden countries in Africa reporting the highest absolute increase in malaria cases in 2018 compared to 2017 [1].

Targeting malaria elimination, mass screening and treatment (MSAT) aims to detect and treat all infections, including asymptomatic *Plasmodium* carriers with low-density infection, to reduce the parasite reservoir [2, 3]. MSAT usually uses a rapid diagnostic test (RDT) as diagnostic tool. RDTs have a sensitivity comparable to field microscopy. While RDTs are limited regarding sensitivity for *Plasmodium falciparum* infections with < 100 parasites/µl and non-falciparum infections [2], polymerase chain reaction (PCR) has a limit of detection as low as 0.02 parasites/µl and enables differentiation of non-falciparum species [2, 4]. Since the proportion of submicroscopic malaria infections depends on the transmission intensity and ranges from 20 to 80%, molecular diagnostic methods are necessary to understand local malaria epidemiology [3].

Despite asymptomatic parasitaemia being increasingly recognized as a major hurdle towards malaria eradication, it has rarely been a major research focus. Especially studies on asymptomatic adults and asymptomatic non-falciparum infections are scarce [5, 6].

The present work reports the results of a survey using both RDT and PCR to assess asymptomatic carriage of *P. falciparum* and non-falciparum species in adult residents of a Ghanaian forest region during the high transmission season in order to inform policy-makers regarding the optimal strategies to reduce malaria burden in Ghana.

## Methods

Adults volunteers were recruited during the second rainy season in September 2018 in five villages in the Asante Akim North district in the Ashanti region, Ghana. The district lies within the moist semi-deciduous forest belt of Ghana. The forest areas are characterized by a tropical climate with two rainy seasons [7]. The villagers live in simple houses constructed from local materials and their main occupation is farming. One to three days before recruitment, community health workers visited the representative villages and invited all adults who lived in the catchment area to participate. Exclusion criteria were clinical signs of infection (axillary temperature ≥ 37.5 °C or history of fever in the past 48 h, headache, chills, myalgia, dizziness, nausea and diarrhoea), pregnancy and puerperium.

On the day of blood collection, field workers informed the villagers again about the exclusion criteria and the planned procedure of blood collection. Participants were only included after providing written informed consent. Age, gender and absence of exclusion criteria were assessed with a questionnaire for each participant. An RDT targeting the histidine-rich protein II antigen specific to *P. falciparum* and a pan-malarial antigen common to *Plasmodium vivax*, *Plasmodium ovale*, and *Plasmodium malariae* was performed on venous blood immediately to inform all participants about their test result (BinaxNOW Malaria Test; Binax, Inc., Scarborough, ME, USA). According to the WHO guideline, faint test bands were interpreted as positive [8]. Similar to a recent study, faint test bands were defined as being only visible in a good light in agreement of two members of the study team observing the test [9]. If the band was difficult to see in good light but both members of the study team still agreed to see it, the test was interpreted as very faint and reported separately.

For blood counts and PCR, venous blood was collected in EDTA blood tubes (Sarstedt). Blood counts were performed the same day using the Sysmex XP-300 automated hematology analyzer (Sysmex, Kobe, Japan). For PCR analyses EDTA-anticoagulated blood was centrifuged and the pellet was collected and stored at − 80 °C. Frozen samples were shipped to the National Reference Centre for Tropical Pathogens, Bernhard Nocht Institute of Tropical Medicine, Hamburg, Germany. Nucleic acid was extracted manually from 200 μl of frozen red blood pellet using the QIAamp DNA Blood Mini-Kit (Qiagen, Hilden, Germany) according to the manufacturer’s instructions. Screening was performed by genus-specific real-time PCR for *Plasmodium* species (RealStar Malaria PCR kit 1.0, Altona Diagnostics, Hamburg, Germany) as described before [10, 11].

Positive samples were subtyped using species-specific real-time PCR targeting *P. falciparum*, *P. vivax*, *P. ovale*, *P. malariae,* and *Plasmodium knowlesi* (Altona Diagnostics, Hamburg, Germany) as described by the manufacturer and others [10, 11]. If the commercial species-specific PCR was negative, a real-time in-house one-tube SybrGreen malaria PCR was performed as described previously. The species-specific in-house PCR targets *P. falciparum*, *P. knowlesi*, *P. vivax*, *P. ovale*, and *P. malariae* and was earlier shown to be more inhibition-resistant compared to the commercial PCR [11]. Furthermore, sub-species identification was performed for all *P. ovale* infections using an in-house PCR according to the protocol of Bauffe et al*.*, as previously described [12]. Data analyses were conducted using R (R Foundation for Statistical Computing, version 3.4.3).

The study was approved by the Committee on Human Research, Publication and Ethics at the Kwame Nkrumah University of Science and Technology, Kumasi, Ghana (CHRPE/AP/455/18).

## Results

A total of 401 participants were screened for symptoms. One person was excluded from the study before blood collection due to dizziness. Therefore, 400 participants were included in the study. In 16 out of 400 samples both malaria PCR and internal control PCR (IC) were initially negative, indicating PCR inhibition. The samples were diluted 1:10 and measured again. After dilution, four of the 16 samples tested positive for *Plasmodium* species, three were negative and nine remained malaria PCR and IC negative and thus were excluded from the study.

**Fig. 1:**
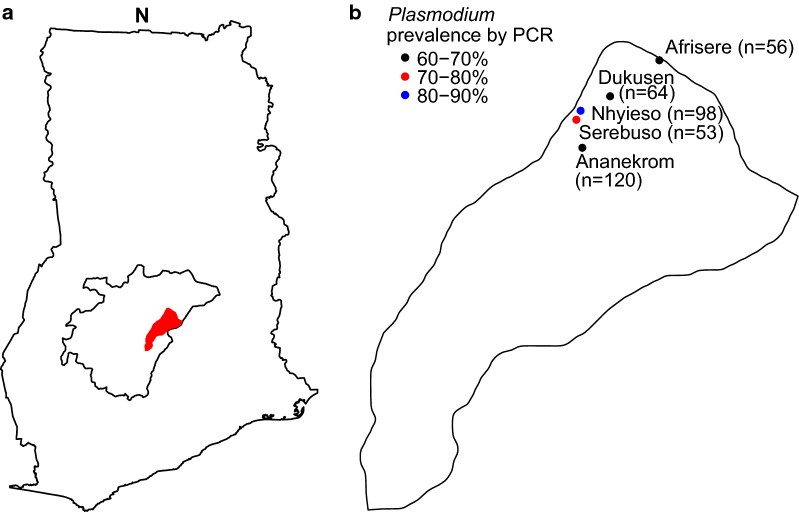
**a** Location of Asante Akim North (red) in the Ashanti region (outlined in black), Ghana. **b** Epidemiological map of study communities in Asante Akim North. The figure was created using the R packages ggplot2, maps and raster

The remaining 391 participants, including 206 women and 185 men, had a median age of 32 years (interquartile range [IQR] 25–44 years). The localization of the study sites in the Asante Akim North district and the range of participants with asymptomatic *Plasmodium* infection among the villages are shown in Fig. 1a, b. Village coordinates are given as previously reported [13]. A total of 284 study participants (73%) tested positive for *Plasmodium* species by PCR. While 266 (68%) participants tested positive for *P*. *falciparum*, 33 (8%) tested positive for *P*. *malariae* and 34 (9%) for *P*. *ovale*. (Table 1). Both *P. ovale curtisi* (3%) and *P. ovale wallikeri* (4%) were identified at similar rates. In seven cases (2%) with high cycle threshold (C_t_) values (low parasite density) sub-species differentiation was unsuccessful. All participants were negative for *P*. *vivax* and *P. knowlesi*.

**Table 1:** *Plasmodium* species detection

*Plasmodium* species detection	PCR (total n = 391)	
	No	%
Pan-*Plasmodium* positive	284	73
*P*. *falciparum*	266	68
Monoinfection	218	56
Coinfection with *P*. *malariae*	22	6
Coinfection with *P*. *ovale*	20	5
Coinfection with *P*. *malariae* + *P*. *ovale*	6	2
*P*. *malariae*	33	8
Monoinfection	5	1
Coinfection	28	7
*P*. *ovale*^a^	34	9
Monoinfection	8	2
Coinfection	26	7
Species not determined	5	1

^a^n = 15 (4%) *P*. *ovale wallikeri*, n = 12 (3%) *P*. *ovale curtisi*, n = 7 (2%) unknown subspecies

**Fig. 2:**
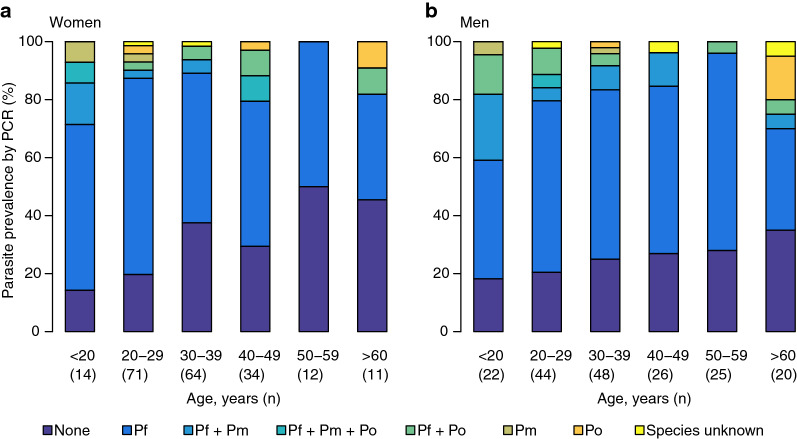
Prevalence of *Plasmodium* infection detectable by PCR among (**a**) women and (**b**) men of different age groups. Pf, *Plasmodium falciparum*; Pm, *Plasmodium malariae;* Po, *Plasmodium ovale*

While *P. falciparum* presented mainly as monoinfection (82%), *P. malaria* and *P. ovale* were observed mainly in the context of coinfections with 85% (28/33) and 76% (26/34), respectively. The distribution of *Plasmodium* infections among different age groups in women and men is demonstrated in Fig. 2a, b.

**Fig. 3:**
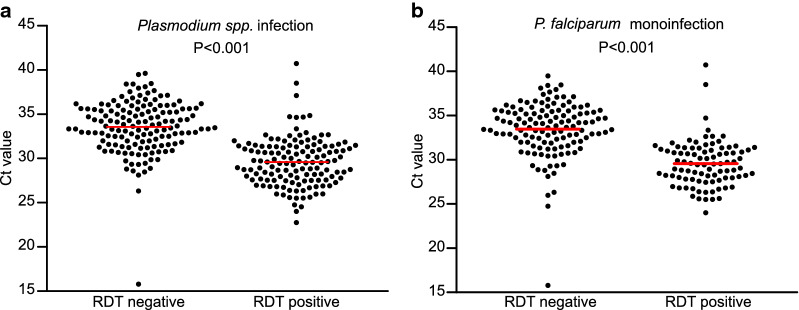
PCR C_t_ values according to RDT result for **a** *Plasmodium* species infection and **b** *P. falciparum* monoinfection. Red lines indicate median values. C_t_, cycle threshold; *P.*, *Plasmodium*; RDT, rapid diagnostic test

RDT only detected *Plasmodium* species in 126 study participants (32%). Adjusted for age, gender and village, the C_t_ value of the commercial screening PCR was significantly associated with a positive RDT in the whole cohort and in the subgroup with *P. falciparum* monoinfection (Fig. 3a, b). Compared to PCR, sensitivity and specificity of the RDT for detection of *Plasmodium* infection were 43% (122/282) and 96% (102/106). When interpreting very faint bands (n = 17) as positive, sensitivity increased to 48% (136/282) and specificity decreased to 93% (99/106).

Participants with *Plasmodium* carriage confirmed by PCR were younger on average (median age, 31 years; IQR, 23–41) compared to participants with negative screening PCR who had a median age of 35 years (IQR, 29–48 years).

Among the parameters measured by blood count, the platelet count was inversely associated with *Plasmodium* carriage status detected only by PCR in both univariate and multivariate analysis, adjusted for age, gender and village (Table 2). Haemoglobin and white blood count did not differ between the groups.

**Table 2:** Predictors of asymptomatic *Plasmodium* infection in univariate and multivariate analyses

Variable	Median (IQR)		Univariate	Multivariate^a^	
	PCR positive	PCR negative	P-value	β	P-value
White blood cells (× 10^9^/l)	5.5 (4.7–6.7)	5.7 (4.6–6.9)	0.741	0.04	0.822
Haemoglobin (mg/l)	13.1 (12.3–14.2)	13.2 (12.3–14.3)	0.747	− 0.14	0.346
Platelets (× 10^9^/l)	225 (183–271)	253 (196–308)	0.004	− 17.94	0.03

For univariate analyses Wilcoxon rank-sum test was used, multivariate analyses were performed using linear regression

^a^Adjusted for age, gender and village

## Discussion

The present study conducted in the rainy season in the forest zone of Ghana reveals a very high prevalence of asymptomatic *Plasmodium* infection, with 73% of adult residents being PCR-positive for at least one *Plasmodium* species. In the studied populations, asymptomatic adults represent a relevant reservoir for malaria parasites. Any attempt at malaria eradication therefore must target a wider population and should not only focus on children or individuals with a positive RDT. In endemic regions, surveys should be conducted on a regular basis.

This is the first study on molecular prevalence of asymptomatic *Plasmodium* infections in adult residents of rural areas in the Ashanti region. A study conducted in 1998 observed a prevalence detected by microscopy of 51% in the forest area of the Ashanti region with a peak among 8-year-old children and a plateau at about 20% in adults [14]. The prevalence of adult asymptomatic parasite carriers assessed by PCR was considerably higher in the present study.

In a study including 160 asymptomatic adults and children from the Greater Accra Region in Southern Ghana, the prevalence of asymptomatic parasite carriers based on microscopy was 34% and 4% in a high and low malaria transmission area, respectively [15]. However, molecular diagnostic tools for detection of submicroscopic parasitaemia were not performed. In the Upper East region of Ghana, which is considered as a part of the Guinea Savannah Zone, asymptomatic *P. falciparum* carriage rates detected by PCR among all age-groups were 14% during the dry season [16]. In the same region, the molecular prevalence of *P. falciparum* infection was 72% in asymptomatic adults > 19 years recruited in the rainy season in 2000 [17]. Among adult residents (> 20 years) of the Guinea Savannah Zone recruited in 2012–2013, the prevalence of asymptomatic *P. falciparum* infection assessed by combined microscopy and PCR was 64% and 27% in the wet and dry seasons, respectively [18].

With 68%, the prevalence of asymptomatic *P*. *falciparum* parasitaemia observed in the present study was, therefore, similar to studies conducted in the rainy season in the Guinea Savannah zone [17, 18] and in a rain forest region in Gabon [19]. In a cross-sectional study performed in the Eastern region of Ghana in 2017, the positivity rate among adults 20 years old and above was 14% by RDT and about 55% by PCR [20]. Despite the fact that this study included febrile cases also, the observed prevalence was considerably lower than in the present survey.

Subtypes of *P*. *ovale* have not been widely reported specifically in asymptomatic Ghanaian adults before. *Plasmodium ovale* was identified in nearly 10% of the study participants, with similar numbers of individuals positive for *P*. *o. curtisi* and *P. o. wallikeri*. The importance of the identification of *P. ovale* is increasingly recognized, since both subtypes are perceived as relapsing malarial parasites, with *P*. *o. curtisi* reappearing in shorter time intervals compared to *P*. *o. wallikeri* [21]. Asymptomatic infections with *P. malariae* were also common with 8% in the present study. Comparable to the study from Gabon [19], non-falciparum infections presented mostly as mixed infections with *P. falciparum*.

Even though the WHO suggests that even faint RDT bands should be interpreted as positive, a recent study concluded that it is possible to interpret faint test bands as malaria negative if the patient does not have risk factors of developing severe malaria [9]. In the present study, 43% of asymptomatic *Plasmodium* species carriers were detected by RDT. Even if very faint bands were interpreted as positive, the sensitivity of the RDT was still less than 50%. In a study on MSAT in Zanzibar, anti-malarial treatment of RDT-positive individuals did not reduce subsequent malaria incidence, compared with control areas. Only 4% of parasite carriers were detected by RDT compared to PCR in that study [2]. In the cross-sectional study performed in the Eastern region of Ghana, about one out of four malaria cases in individuals aged ≥ 20 years, which were detected by PCR, were also identified by RDT [20].

The considerable variance in the sensitivity of RDTs among different studies highlights that molecular diagnostic tools are necessary to adequately assess the malaria prevalence and improve MSAT programmes.

## Conclusion

Asymptomatic parasitaemia in adults, including cases with non-falciparum species, constitutes a relevant reservoir for transmission in the Ghanaian forest zone and must be considered in efforts towards elimination of malaria.

## Data Availability

The datasets used and/or analysed during the current study are available from the corresponding author on reasonable request.

## Abbreviations

C_t_: Cycle threshold
IQR: Interquartile range
MSAT: Mass screening and treatment
PCR: Polymerase chain reaction
RDT: Rapid diagnostic test
WHO: World Health Organization
